# A dataset on the contents of 100 terms of service of online platforms, analyzed and evaluated under the EU consumer law

**DOI:** 10.1016/j.dib.2024.110136

**Published:** 2024-02-01

**Authors:** Przemysław Pałka, Radosław Pałosz, Andrzej Porębski, Katarzyna Wiśniewska

**Affiliations:** Faculty of Law and Administration, Jagiellonian University, 12 Bracka St., 31-005 Krakow, Poland

**Keywords:** Digital economy, Contract, Regulation, Unfair clauses

## Abstract

The dataset contains information obtained during the analysis and evaluation of the contents of 100 Terms of Service (ToS) of online platforms from the point of view of the European Union consumer law. Each ToS has been assigned information regarding the presence and quality of remedy clauses, dispute resolution clauses, unilateral alteration clauses, rights to police the behavior of users, and regulatory requirements. In addition, descriptions of service features and parties’ rights and duties have been pulled out.

The sample contains 100 ToS of digital platforms operating in sixteen market sectors: Cloud storage, Communication, Dating, Finance, Food, Gaming, Health, Music, Shopping, Social, Sports, Transportation, Travel, Video, Work, and Various. The selected companies’ headquarters span four legal surroundings: the US, the EU, Poland specifically, and other jurisdictions (including, e.g., China, Japan, and the UK). The chosen platforms are both privately held and publicly listed and offer both fee-based and free services. The resulting data table includes 100 observations described by 38 variables (10 metadata and 28 presenting results of the analysis). The definitions of variables and categorical values are presented in the instruction followed by the annotators. All the analyzed ToS in original and annotated form are a part of the dataset.

The documents were retrieved from publicly accessible websites of respective online platforms on February 22, 2022, from the territory of Poland, the European Union. Each document was subsequently annotated independently by two researchers, based on the enclosed instruction. The instruction was prepared by the PI, with the help of the team, based on the EU law. The annotators subsequently run consistency checks. The process was designed to ensure the lack of errors and the clarity of the instruction. When ambiguities in the latter were discovered, the PI and the annotators resolved them, and the previously tagged documents were retroactively examined for consistency.

These data have significant reuse potential. They can be reused by social scientists attempting to understand the dynamics of the digital markets or normative scholars, like lawyers or political philosophers, attempting to create algorithms for scoring online consumer contracts. They can also be reused by various non-scholarly actors, including policymakers verifying the efficacy of their regulations, developers willing to market their products in a consumer-friendly way, or consumer organizations attempting to raise consumer awareness. The data is suitable for many different types of data analysis methods, such as cluster analysis, dimensionality reduction, classification methods, and scoring.

Specifications Table

**Table:** 

Subject	Law
Specific subject area	Consumer law, empirical contracts, online platforms, consumer-friendliness
Data format	Raw and Analyzed
Type of data	Table, Text, Graph
Data collection	The documents were retrieved from publicly accessible websites on February 22, 2022, from the territory of Poland, the European Union. Each document was subsequently annotated independently by two researchers, based on the enclosed instruction. The instruction was prepared by the PI, with the help of the team, based on EU law. The annotators subsequently ran consistency checks. When ambiguities in the latter were discovered, the PI and the annotators resolved them, and the previously tagged documents were retroactively examined for consistency. The results were represented in the Microsoft Excel file.
Data source location	Total of 100 Terms of Service was collected on February 22, 2022, from publicly accessible websites, in versions binding for consumers residing in the European Union, from the territory of Poland.
Data accessibility	Repository name: Mendeley Data Data identification number: doi: 10.17632/dtbj87j937.3 Direct URL to data: https://data.mendeley.com/datasets/dtbj87j937/3

## 1 Value of the Data

• These data are valuable because they provide rich insight into the contents of the Terms of Service of online platforms operating in 16 market sectors.
• They are also valuable because they allow for comparison of contents based on several criteria, including the corporation's jurisdiction of origin, whether the service is free, or whether the company is publicly listed.
• These data can benefit law and policy scholars trying to assess the efficacy of government regulation of consumer contracts online. It can also benefit governments themselves, reflecting upon the efficacy of various regulatory approaches.
• The data are suitable for many different types of data analysis methods, such as cluster analysis, dimensionality reduction, classification methods, and scoring. They can be used for both research and teaching purposes.
• These data can be reused by social scientists attempting to understand the dynamics of the digital markets and normative scholars, like lawyers or political philosophers, attempting to create algorithms for scoring online consumer contracts.
• They can also be reused by various market participants, including developers willing to market their products in a consumer-friendly way, as well as consumer organizations attempting to raise consumer awareness.

## 2 Data Description

This article describes a repository that holds a dataset generated from a study of 100 Terms of Service (ToS) governing the functioning of various online services [1]. The contents of these ToS were reconstructed and evaluated under the EU consumer law [2], [3], [4], [5]. The repository consists of the following files:

(1) table “Terms of Service Analysis and Evaluation_RESULTS” with the results of ToS analysis (this table has .xlsx and .csv versions);
(2) table “Variables Definitions.xlsx” with the tagging instruction, including definitions of variables and categorical values in the results table;
(3) set of 100 raw ToS (“Clear ToS” folder and .pdf files inside it);
(4) set of 100 ToS read and annotated independently by two researchers, from which the final results were retrieved (folder “Annotated ToS” with subfolders “Annotator 1” and “Annotator 2” and .docx files inside them).

The main dataset is stored in the file titled “Terms of Service Analysis and Evaluation_RESULTS,” uploaded both in .xlsx and .csv formats. These versions are fully comparable, but .xlsx has some additional text formatting for simpler use of data in the spreadsheet, hence we recommend using the .xlsx version for using data inside a spreadsheet and .csv version for use in statistical environments and other programs. The dataset contains information about the contents of the ToS. Each document has been described using the following variables:

1. Metadata (10 variables): These variables provide information about the document with ToS, the services, and the companies offering them, including: 1.1. the name of the service (name of variable: *name*); 1.2. URL from which the ToS has been downloaded (*url*)*;* 1.3. the effective date of the ToS (*date*)*;* 1.4. the language in which ToS was written; 1.5. the sector in which the company operates (*sector*)*;* 1.6. number of words in the document *(word_cnt*); 1.7. the jurisdiction of the service provider's main headquarters or the headquarters of the top-parent company of the company providing the service, if the service provider has a parent company (*hq*); 1.8. the higher-level category of the jurisdiction (*hq_cat*); 1.9. whether the company or the top-parent company (if exists) is publicly listed or privately held (*public*); 1.10. whether the service is paid or free (*paid*)*.* Some clarificatory remarks on definitions of some metadata variables can be found in the “Sample” heading in the next section of the article.
2. ToS Evaluation Results (Evaluative Variables) (24 categorical variables with ordinal interpretation): These variables are used to store the results of the evaluation of each ToS using the values −1, 0, and 1, based on specific criteria tailored for each variable, specified in the file titled “Variables Definitions.” They include: limitation of remedy clauses (2.1 – 2.5); dispute resolution clauses (2.6 – 2.9); unilateral alteration clauses (2.10 – 2.14); right to police the behavior of users (2.15 – 2.16); regulatory requirements (2.17 – 2.19); and various (2.20 – 2.24). If a particular variable is irrelevant for a specific ToS, the corresponding cell is filled with “NA”. General rule for assigning values: −1 represents solutions unfavorable to the consumer (under the EU law), 1 most favorable, and 0 is an intermediate value.
3. Count Variables (2 count variables): These variables represent a number of occurrences of certain types of clauses in each ToS. They include: 3.1. the number of clauses seen as unclear (*uncle*) and 3.2. the number of documents referred to by the ToS (*docu*).
4. Pull-out Text Variables (2 text variables stored in 10 columns): These variables contain excerpts from the ToS. They include: 4.1. rights and obligations of the parties (*core*) and 4.2. descriptions of the service (*what*). These variables appear in several (3 and 7, respectively) columns consecutively, to keep each descriptive instance separate.

The second file, called “Variables Definitions,” provides descriptions of each variable, explaining how the relevant clauses in the ToS were tagged. It is a table divided into 4 sheets, each dedicated to a group of variables. The first sheet, “Evaluative Variables,” offers instructions for assigning values to the numerical variables. It consists of 5 columns. The first one contains the names of the variables, which mark the issues relevant for assigning a value under each category. The second column, “Legal ground”, cites the legal basis used to construct the instructions, mailny rooted in the European Union law. The next column presents numerical codes that were used to assign values in “Terms of Service Analysis and Evaluation_RESULTS” and in the text of the analyzed documents. The last two columns of the table present detailed instructions on how to assign each score to ToS. Besides precise explanations on how to value a particular ToS, these instructions contained also disambiguations for possibly unclear terms, like “valid”. They were also prepared for the researchers without the legal background, clearly explaining the more advanced terms functioning in legal practice.

The second sheet, “Count Variables,” includes the names, codes, and descriptions of variables used to count the occurrences of specific clauses in a given ToS. The third sheet in the “Variables Definitions” file presents the pull-out variables, providing their names, codes, and short descriptions. The fourth sheet, “Metadata,” describes the metadata variables and indicates which information about the service should be extracted to the “Terms of Service Analysis and Evaluation_RESULTS”. It also clarifies any potential doubts about the meaning of legal or general terms.

The two folders, “Clear ToS” and “Annotated ToS” contain the sources from which data has been extracted. The former includes the 100 contracts in the .pdf formats, retaining the original formatting. The latter is further divided into two subfolders: “Annotator 1” and “Annotator 2.” They contain .docx files with the text of each analyzed ToS, with the annotations. Both folders include a copy of every analyzed ToS. The files within each folder were analyzed separately by the two annotators. They read the ToS and marked the relevant clauses based on the definitions described in the “Variables Definitions” file. The presence of both subfolders allows for the comparison of the work done by the taggers, enabling verification of the reliability of consistency checks performed throughout the study.

To show the general characteristics of the set and the possible ways to analyze its various variables, the distributions of several evaluative variables will be shown and compared, and the count variables compared in terms of *hq_cat*. The analysis in this work was performed using JASP ver. 0.17.2.1 [6] and the statistical environment R ver. 4.2.2 including the *tidyverse* package [7].

**Fig. 1:**
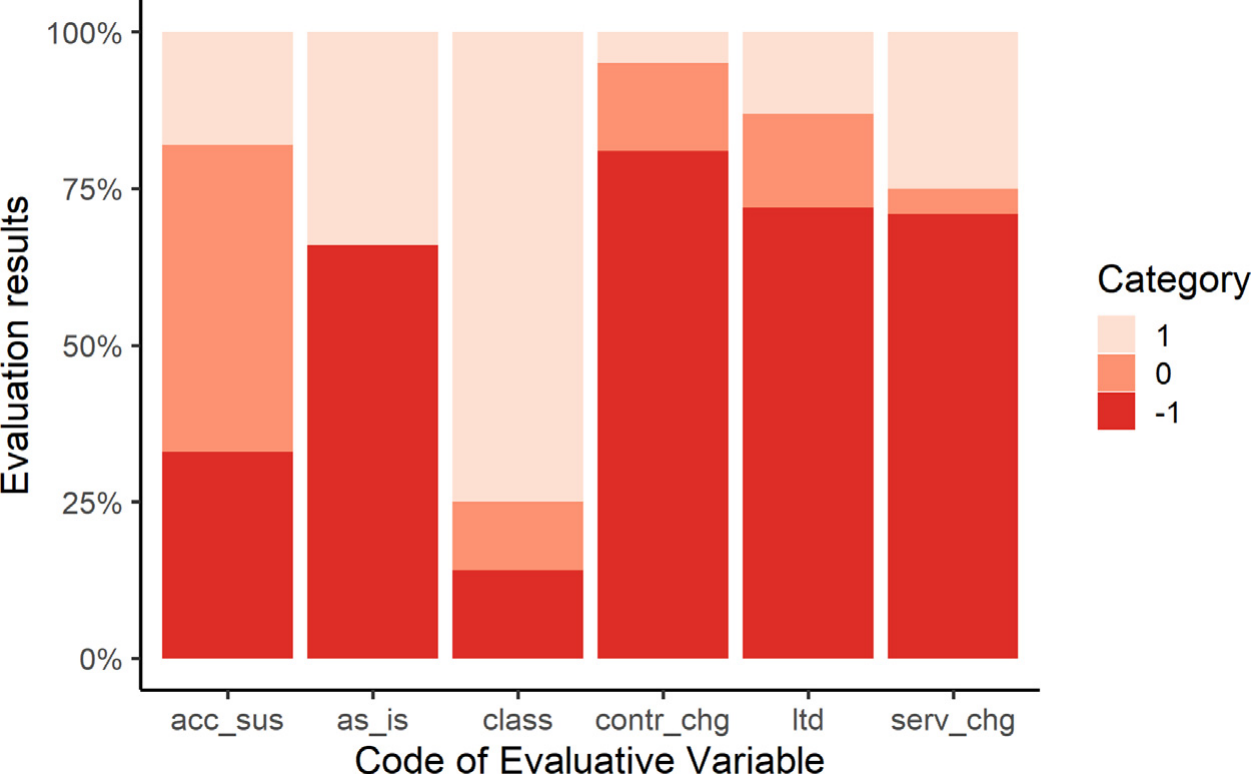
Evaluation results of six Evaluative Variables: *acc_sus, as_is, class, contr_chg, ltd, serv_chg*.

The evaluation results for the six selected variables (*acc_sus, as_is, class, contr_chg, ltd, serv_chg*) are presented in Fig. 1. As can be seen, ToS are more or less positively evaluated under different variables. The most positively rated of the six is the *class* variable, which refers to existence of class action waivers, present in some of the contracts. They prevent the consumers from joining class action lawsuits, which allow similar claims to be asserted by a large group of people in a single proceeding.

**Fig. 2:**
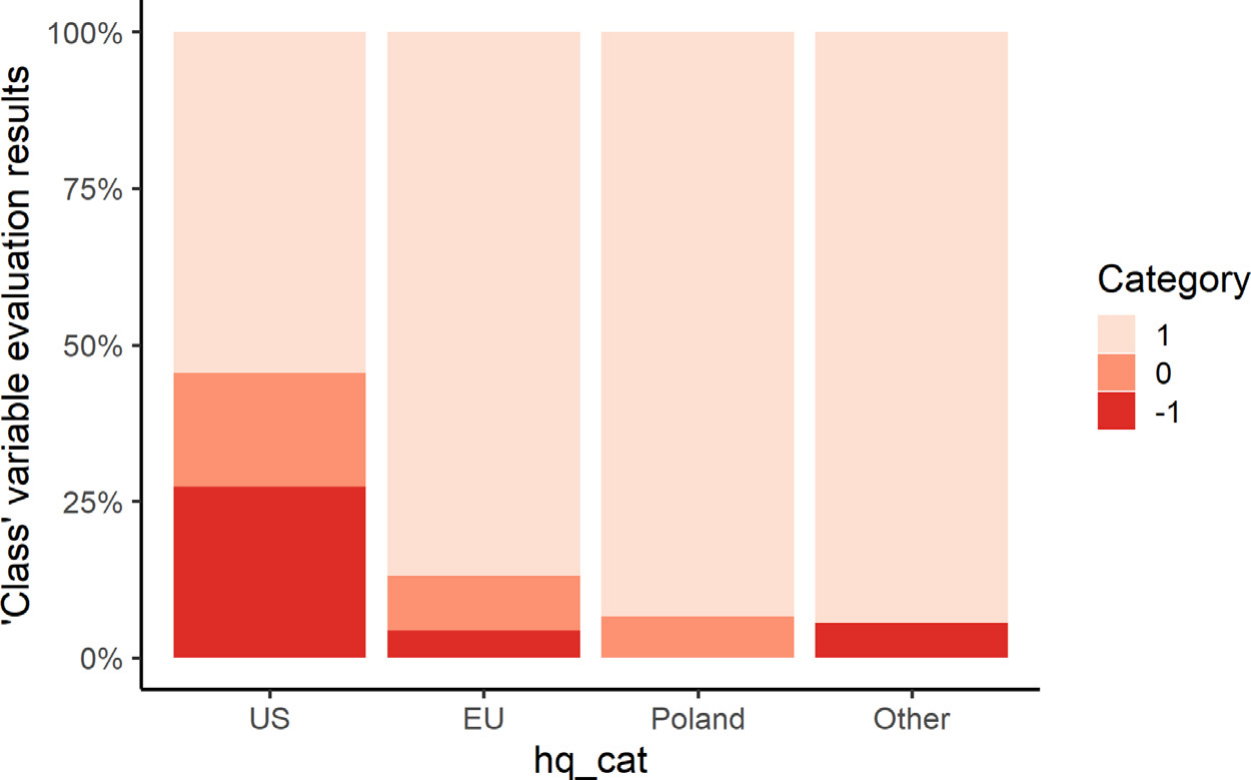
Evaluation results for “class” variable, grouped by *hq_cat*.

Using the example of *class* variable, we show the possibility of comparative ToS analysis between jurisdictions, see Fig. 2. Fisher's Exact Test comparing the values assigned to this variable in different jurisdictions reveals statistically significant differences, resulting from a higher percentage of lower ratings for the US (*p* = .004).

The ability to analyze count variables will be shown on both variables of the number of references to other documents (*docu*) and the number of unclear clauses (*uncle*) in the ToS. In both cases, the variables will be compared between jurisdictional categories.

**Fig. 3:**
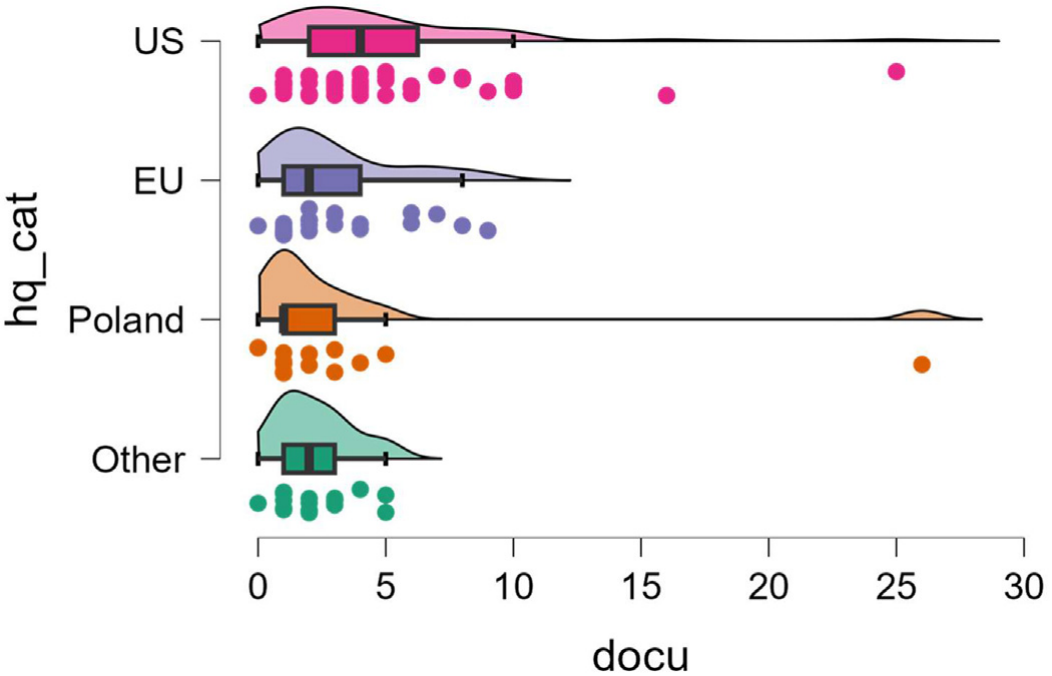
Distribution of the number of references to other documents in ToS (*docu*) depending on region of jurisdiction (*hq_cat*).

The variable *docu* takes values in the range 0–26, with median = 3, 1st quartile = 1 and 3rd quartile = 5. Poisson regression model indicates statistically significantly higher values of this variable for US, compared to other countries (*p* = .013 in comparison with Poland, *p* < .001 for other comparisons). Detailed distributions of the variable for each jurisdiction can be seen at the raincloud in Fig. 3.

**Fig. 4:**
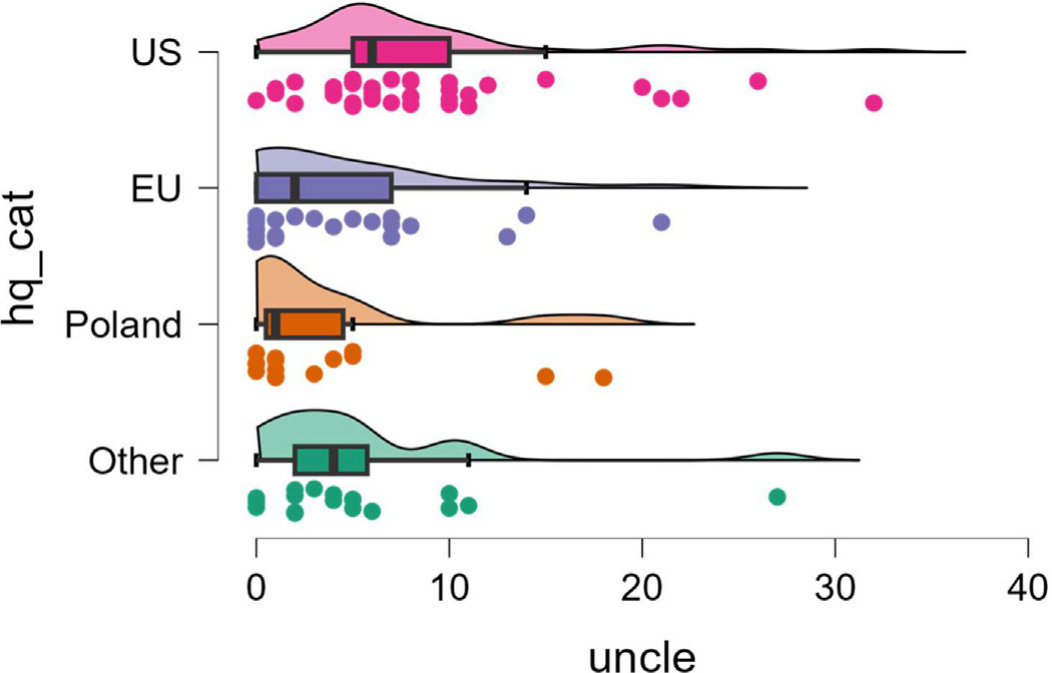
Distribution of the number of unclear clauses in ToS (*uncle*) depending on region of jurisdiction (*hq_cat*).

The variable *uncle* takes values in the range 0–32, with median = 5, 1st quartile = 1 and 3rd quartile = 8. The ToS for the US had the highest values for the variable, as implied by the Poisson regression model (*p* < .001 for comparison with each other jurisdiction category). For a more detailed exploration of the distributions of this variable in different *hq_cat* groups, see Fig. 4.

## 3 Experimental Design, Materials and Methods

### 3.1 Purposive sampling

The set of all online platforms remains indeterminable due to a lack of an obligatory registry for such operations. Therefore, it is not possible to construct a strictly understood statistical frame that could be used to collect a random sample of online platforms. Although some of these entities operate as Small to Medium Enterprises (SMEs), exclusively catering to their domestic consumer base, a substantial portion are multinational corporations that function in numerous markets, necessitating universal operational standards that obviate the need for adjustment to specific national legal mandates. Since the world's largest platforms such as Google, Facebook and YouTube have the most users and the largest share of network traffic [8], a purposive sample including such large platforms will allow the study of those digital entities with which the most Internet users interact. These prevailing conditions and assumptions have driven us to adopt purposive sampling [9]. The primary focus was on covering the vast majority of the largest social media platforms, defined in terms of their reach – specifically, the number of monthly active users (e.g., Meta's services – Facebook or Instagram – or YouTube) [10,11], and many of the products of the largest Internet companies operating in Poland, defined in terms of their market capitalization (e.g., Amazon or Adobe) [12]. This criterion ensured the inclusion of platforms that have significant user base and are broadly recognized.

Additionally, the selection of platforms was made with the intention to encompass all the predefined market sectors and high-level jurisdictions (the EU, Poland specifically, the US, and Other). This choice was critical to ensure the dataset's comprehensiveness, heterogeneity, and relevance across different market contexts. Inclusion of both SMEs and larger companies was another key consideration in this process, aimed at capturing the diversity of online platforms in terms of their operational scale. When deciding on the final inclusion of platforms in the sample, their prominence in Google search results was also taken into account. As Google is the most relevant search engine on the Internet, this method provided a proxy to assess the platforms’ popularity.

In the context indicated, the presented dataset, as selected purposively, can be used to draw conclusions for the subpopulation of platforms most widely recognized globally and locally (in Poland), but not to infer the (by the way, unknown) population of all online platforms [13].

### 3.2 Inclusion guidelines

The inclusion guidelines for selecting the 100 Terms of Service (ToS) of digital platforms based on the provided information are as follows:

1. Market Sectors:
  • The platform should belong to one of the sixteen distinct market sectors: Cloud storage, Communication, Dating, Finance, Food, Gaming, Health, Music, Shopping, Social, Sports, Transportation, Travel, Video, Work, or the “Various” category.
2. Geographical/Legal Surroundings:
  • The goal is to, where possible, have representation from at least one company from each of the four legal surroundings within each market sector.
3. Representativeness for a typical Internet user:
  • The overall selection aims to provide a comprehensive representation of the market. This assumes that there should be a balance across sectors, legal surroundings, corporate structures, and service offerings, with a preference for widely recognized global and local (in Poland) online platforms in the sample. Therefore, the criterion for inclusion was to increase the diversity of the sample while having a high estimated recognition of the platform.

### 3.3 Sample

We have chosen 100 Terms of Service of digital platforms, operating in sixteen distinct market sectors: (1) Cloud storage, (2) Communication, (3) Dating, (4) Finance, (5) Food, (6) Gaming, (7) Health, (8) Music, (9) Shopping, (10) Social, (11) Sports, (12) Transportation, (13) Travel, (14) Video, (15) Work and (16) Various category, see Table 1. The selected companies’ headquarters span four legal surroundings: the United States (44 entities), the European Union (23), Poland specifically (15), and other jurisdictions (18), see Table 2. Detailed list of countries in *Other* group is included in Table 3. The chosen array of digital platforms incorporates a diverse range of corporate structures, including both privately held enterprises (39 entities) and those publicly (38) or indirectly publicly (23) traded on stock exchanges. Additionally, it covers a spectrum of service offerings that span both fee-based (28), optionally paid (64), and complimentary services (8), thereby providing a comprehensive representation of the market.

**Table 1:** Frequencies of *sector* variable.

*sector*	Frequency	*sector*	Frequency	*sector*	Frequency	*sector*	Frequency
Cloud storage	5	Food	6	Shopping	6	Travel	8
Communication	7	Games	8	Social	10	Various	6
Dating	5	Health	4	Sport	6	Video	7
Finance	7	Music	2	Transport	7	Work	6

**Table 2:** Frequencies of *lang, hq_cat, publ, and paid* variables.

*lang*	Frequency	*hq_cat*	Frequency	*publ*	Frequency	*paid*	Frequency
ENG	87	EU	23	Private	39	Free	28
PL	13	Poland	15	Indirectly public	23	Optionally paid	64
		US	44	Public	38	Paid	8
		Other	18

**Table 3:** Frequencies of particular countries in *Other* group in *hq* variable.

*hq*	Frequency	*hq*	Frequency	*hq*	Frequency
China	6	India	1	Turkey	1
Japan	4	Israel	1	UAE	1
UK	3	Singapur	1		

The process for selecting sectors was a collaborative effort, primarily driven by discussions among the research team. The first step in choosing sectors involved identifying those to which the selected large platforms belonged. Subsequently, the selection of sectors was expanded based on the expert knowledge of the research team. This expansion was twofold: firstly, it included sectors chosen for their unique characteristics or potential specificity, such as the health sector. Secondly, the categorization of applications in popular app stores, like the App Store and Google Play was considered. This approach allowed the research team to align sector selection with contemporary consumer usage patterns and the digital marketplace's categorization. In determining the final categorization of platforms (*sector* variable) the platform's main functionality, its market impact, representation within a sector, and the consensus reached through expert discussions among the research team were considered.

The categorization of services under *paid* variable took into consideration the nature of the free options provided by these services. *Free* services category was specifically assigned to services that were not time-restricted and entirely free. Those that offered a basic version without any time limit but with the possibility of upgrading were considered *optionally paid*. Services that were either fully paid or offered a free trial followed by a paid subscription were classified as *paid* services.

Variables *hq* and *hq_cat* are used to denote the jurisdiction of the headquarters of the service provider company or the top-parent company's headquarters, if any parent company exists. The headquarters information was sourced from Crunchbase [14] and verified using Google Search. While the analysis focused on the content of ToS available in Poland, these variables specifically indicated the location of the company's (or parent company's) headquarters. The direct party to the contract with the consumer, particularly if it's a subsidiary created for EU operations, was *not* the focus of the study. Similarly, the *public* variable is referring to the top-parent company, if any parent company exists.

### 3.4 Location and language

**Fig. 5:**
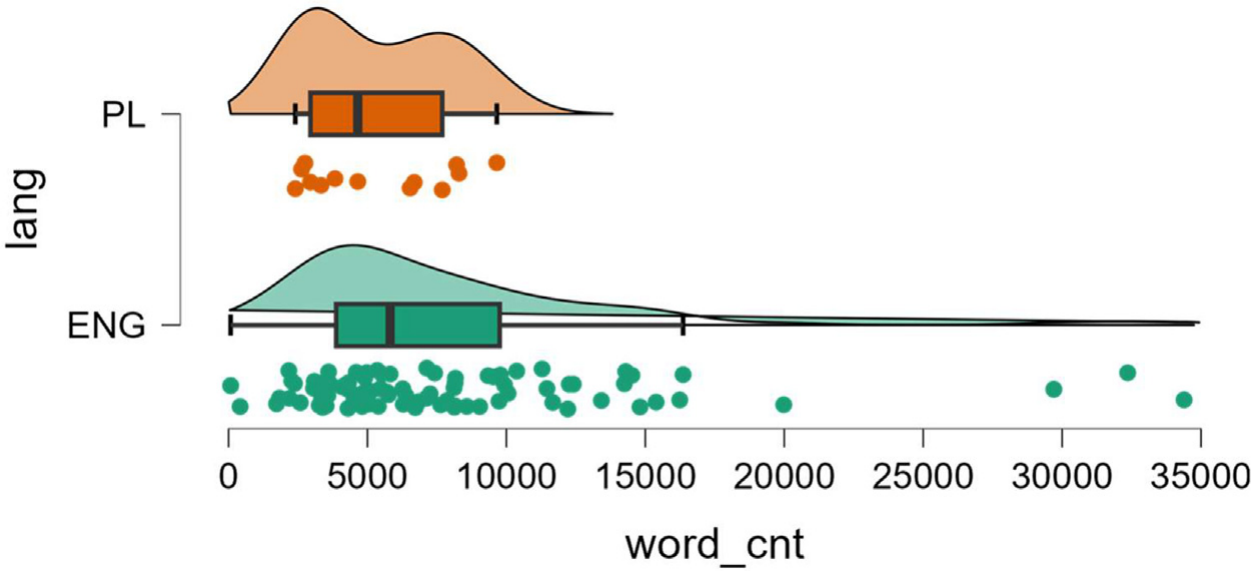
Distribution of the number of words in document (*word_cnt*) depending on document language (*lang*).

The total count of one hundred ToS was retrieved from the publicly accessible websites of respective online platforms on February 22, 2022, within the geographical confines of Poland. Each of these sets of terms held the potential for applicability by a consumer domiciled in the European Union, specifically Poland. Consequently, they embody the specific versions that platform operators have chosen to use within this legal jurisdiction. The majority of analyzed ToS were written in English (87 entities) with rare exceptions, where only the Polish version was available (13 entities) in case of services exclusively catering to the Polish consumer base. Documents containing ToS varied greatly in length (range: 75–34391), which, however, averaged several thousand words (*M* = 7481, SD = 5880, 1st quartile: 3594, median: 5787, 3rd quartile: 9392). The length of the documents was not statistically significantly different depending on their language (Wilcoxon test's *W* = 698, *p* = .176), but clearly the longest documents, outliers, were in English, see Fig. 5.

### 3.5 Conducting research

The analyzed terms of service were downloaded in Portable Document Format (PDF) and later reformatted using Adobe Acrobat to .docx format for their compatibility with Microsoft Word, facilitating the tagging of pertinent clauses by two researchers utilizing the “comments” feature. Further, the values derived from the annotated clauses were subsequently inserted into the Microsoft Excel file – “Terms of Service Analysis and Evaluation_RESULTS” – for further analysis.

The preliminary framework for the coding system in the study was initially developed based on the applicable law. The aim was to encompass comprehensively all the possible rules governing the ToS, in the EU, at the time of the study, as well as the legal acts yet not applicable though on track of being adopted, like the Digital Services Act. Subsequently, the content of the documents included in the sample allowed the selection of categories to be specified more precisely. The initially established set of categories, grounded in the applicable or forthcoming law, was thus combined with general knowledge and understanding of the ToS to achieve the first version of tagging instruction. As the study progressed, the coding system underwent iterative reconstruction. This involved the codes and variables being refined and adjusted as deeper insights were gained from the sample documents, ensuring they accurately reflected the nuances and specificities encountered in the ToS.

During the intial phases of the study, two annotators independently examined each of the procured documents. Relevant clauses were tagged by assigning them a value based on the predefined criteria. Upon completing a document's analysis, the annotators inscribed these values into the corresponding row of the chart in the “Terms of Service Analysis and Evaluation_RESULTS” file. The tagging process lasted from February 22, 2022, until September 2, 2022.

Following the independent data entries into their respective chart copies, the annotators subsequently engaged in consistency and correctness checks. This process also ensured a uniform interpretation of the “Variables Definitions,” thereby minimizing potential inconsistencies or discrepancies in the variables’ definitions and the data interpretation. In the scenarios where the discourse yielded no definitive resolution, the ambiguity was consulted with the Principal Investigator (PI), who gave final decision on the classification of the given case and, whenever necessary, clarified the ambiguity in the instruction. These conflicts were minor and primarily stemmed from the ambiguity present in the ToS themselves and concerned only particular provisions. When such clarifications were made, the previously tagged documents were retroactively examined and modified to achieve consistency with the newly established criteria. The consistency and correctness of the annotation were ensured through discussions conducted among the research team. When discrepancies appeared in the initial annotations, they were resolved through collaborative discussions until a consistent and unanimous opinion was reached by all members of the research team. This method ensured that the final annotations resulted from consensus, reflecting a shared understanding and interpretation of the data by the entire research team. The consistency verifications were implemented for Evaluative Variables. There was a total of 11 consistency checks, that took place on the following dates: 18.02.22, 14.03.22, 11.04.22, 27.04.22, 04.05.22, 13.06.22, 08.08.22, 22.08.22, 26.08.22, 29.08.22 and 02.09.22. Formal measures of annotators’ agreement, such as Cohen's kappa coefficient, were not calculated in the study.

When disparate clauses governing the same subject matter were assigned varying values (for example, several different limitation liability clauses featured in the same ToS), the lowest value among them was recorded in the chart. This methodology was adopted to ensure the most conservative approach to recording the data.

## Limitations

Five limitations of this dataset should be made clear. First, these data are not a representative sample of the entire population of online platforms. However, they do concern a large number of the most popular consumer services and make up a relatively large, heterogeneous set. Second, the data concerns contracts as available to consumers in the European Union (specifically Poland), and some contracts might differ in content in other jurisdictions. Yet, most analyzed platforms are global and offer uniform contracts all around the world. Third, data has been collected on February 22, 2022, and some contracts might have been updated by now. However, they allow for historical comparison, nevertheless. Fourth, the assessment of clauses, albeit based on an instruction derived from the positive law, is inherently qualitative in nature [15,16]. It is theoretically possible that different annotators could judge certain clauses differently. For this reason, we include all the annotated ToS as part of the dataset. Finally, the distinction between market sectors is not clear-cut: especially, one platform, i.e., Uber, could be classified as both transport and food delivery.

## Ethics statement

Data does not concern human or animal subjects. It pertains to publicly available contracts governing the usage of online platforms (but not data collected *from* social media platforms). The authors have read and followed the *ethical requirements* for publication in Data in Brief.

## CRediT authorship contribution statement

**Przemysław Pałka:** Conceptualization, Methodology, Validation, Writing – original draft, Writing – review & editing, Supervision, Project administration, Funding acquisition. **Radosław Pałosz:** Validation, Investigation, Data curation, Writing – original draft, Writing – review & editing. **Andrzej Porębski:** Conceptualization, Methodology, Formal analysis, Resources, Data curation, Writing – original draft, Writing – review & editing. **Katarzyna Wiśniewska:** Conceptualization, Methodology, Formal analysis, Resources, Data curation, Writing – original draft, Writing – review & editing.

## Data Availability

Annotated Terms of Service of 100 Online Platforms (Original data) (Mendeley Data).
